# Chloroformization

**Published:** 1874-11

**Authors:** 


The State of the Pupil in Chloroform Anæsthesia as an Indication of Danger.—M. Budin, interne of the hospitals, in examining with care all the details of chloroformization, with the end of discovering some sign which may guide the surgeon in its administration, has observed that there exists a certain relation between the state of the pupils and the more or less profound anæsthesia of the subject. * * *

1. There exists in the surgical anæsthesia, produced by chloroform, a constant relation between the state of the pupil and the period of anæsthesia.

2. During the period of excitation the pupil is dilated.

3. This period passed, the pupil contracts; its atresia, very marked for several minutes, accompanies, in general, complete anæsthesia.

4. Dilatation of the pupil supervening during an operation indicates in general that anæsthesia is less profound, and that return to sensation is near.

5. The state of the pupil may then serve as a guide in the administration of chloroform.

6. During operations of long duration, if the patient is to be kept completely insensible and immobile, the pupils must be kept constantly contracted.

7. Finally, efforts at vomiting may produce dilatation of the pupils, dissipate the insensibility, and awaken the patient; they annihilate in part the effects of anæsthesia.—*Progres Medical—Gaz. des Hopitaux, Oct.* 1, 1874.

